# Ectopic expression of citrus UDP-GLUCOSYL TRANSFERASE gene enhances anthocyanin and proanthocyanidins contents and confers high light tolerance in Arabidopsis

**DOI:** 10.1186/s12870-019-2212-1

**Published:** 2019-12-30

**Authors:** Muhammad Junaid Rao, Yuantao Xu, Yue Huang, Xiaomei Tang, Xiuxin Deng, Qiang Xu

**Affiliations:** Key Laboratory of Horticultural Plant Biology (Ministry of Education), Key Laboratory of Biology and Genetic Improvement of Horticultural Crops (Ministry of Agriculture), Huazhong Agricultural University, Wuhan, Hubei 430,070 People’s Republic of China

**Keywords:** Citrus, proanthocyanidins, total flavonoid contents, anthocyanin, photoprotective, Arabidopsis

## Abstract

**Background:**

Citrus fruits are consumed freshly or as juice to directly provide various dietary flavonoids to humans. Diverse metabolites are present among Citrus genera, and many flavonoids biosynthetic genes were induced after abiotic stresses. To better understand the underlying mechanism, we designed experiments to overexpress a *UDP-GLUCOSYL TRANSFERASE* gene from sweet orange (*Citrus sinensis*) to evaluate its possible function in metabolism and response to stress.

**Results:**

Our results demonstrated that overexpression of *Cs-UGT78D3* resulted in high accumulation of proanthocyanidins in the seed coat and a dark brown color to transgenic Arabidopsis seeds. In addition, the total contents of flavonoid and anthocyanin were significantly enhanced in the leaves of overexpressed lines. Gene expression analyses indicated that many flavonoid (flavonol) and anthocyanin genes were up-regulated by 4–15 folds in transgenic Arabidopsis. Moreover, after 14 days of high light stress, the transgenic Arabidopsis lines showed strong antioxidant activity and higher total contents of anthocyanins and flavonoids in leaves compared with the wild type.

**Conclusion:**

Our study concluded that the citrus *Cs-UGT78D3* gene contributes to proanthocyanidins accumulation in seed coats and confers tolerance to high light stress by accumulating the total anthocyanin and flavonoid contents with better antioxidant potential (due to photoprotective activity of anthocyanin) in the transgenic Arabidopsis.

## Background

Citrus is mostly cultivated fruit crops worldwide and its fruits are consumed freshly or as juice (oranges and mandarins). The fruit provide abundant amount of vitamins, carotenoids, folate, dietary fibers, flavonoids [1], proanthocyanidins (PAs) and anthocyanins [2] and is becomes a direct source of dietary metabolites. Flavonoids are further divided into various subclasses such as flavanones, isoflavones, flavonols, flavones, flavanols and anthocyanins [3] which play important role in the protection and defense of plants against biotic and abiotic factors [4, 5]; in addition flavonoids also reduces the risk of many chronic diseases such as cancer in humans [6, 7].

Proanthocyanidins (condensed tannins) are oligomeric or polymeric flavonoid compounds [8]. PAs are widely distributed in whole plant kingdom but mostly they are accumulated in seeds and fruits [8]. PAs functionally protects plants from abiotic (UV radiation) and biotic (fungus and insect pests) factors [9] and also beneficial for humans (anticancer, anti-inflammatory activity and protect skin from sun damage) [8, 10]. Anthocyanins are also subclass of flavonoids which is involved in pollen dispersal, fruits and flower colors, plant defense, and development [11]. The chemical structures of anthocyanins and flavonols are supposed to have strong antioxidant properties which are vital for scavenging of reactive oxygen species (ROS) [11]. These compounds protect plants from various environmental factors such as intense light, cold, UV damage, high temperature and biotic stresses [12]. Recently, it was reported that several flavonoid pathway genes (o-methyl transferase, flavonol synthase, flavonoid 3′-monooxygenase, leucoanthocyanidin dioxygenase etc.) that belongs to flavone, flavonol and anthocyanin biosynthesis pathways were significantly up-regulated in citrus after Huanglongbing invasion [13–16]. Flavonoid compounds are famous due to antimicrobial, photoprotective and have resilient antioxidant properties to scavenging the ROS abiotic [17, 18] and biotic stress [19]. Whenever citrus plants are exposed to different abiotic and biotic stresses then they stimulate its secondary defense machinery (such as anthocyanins and flavonoids) to protect itself from challenging environmental conditions.

In citrus, many studies have been reported at genetic [20] and anatomical level to understand the tolerance and defense mechanism [21, 22]. Many researches have been reported that citrus plant accumulates secondary metabolites (flavonoids, anthocyanin, etc.) after abiotic stress [23] or biotic stress [19]. However, the key gene to bridge the secondary metabolite and defense response are less known [7]. The Citrus genera consist of about 162 species [24]; of them sweet oranges, mandarins, pummelos, lemons and grapefruits are the famous cultivated Citrus species worldwide. Varied degrees of anthocyanin contents are present among Citrus genera; some wild citrus species possess high level of anthocyanin in fruit, flower and tender leaves [2].

In plants, UDP-glycosyl transferases (UGTs) is a large gene family such as the model plant *Arabidopsis thaliana* contains a total number of 120 UGTs [25]; that is involved in the secondary metabolites biosynthesis, conjugating hormones, abiotic and biotic stress toxins and also influence on plant cellular homeostasis [25]. In addition, some UGTs gene such as UGT74B1 enzyme functions in glucosinolate biosynthesis in Arabidopsis [26]. The UGTs, especially GT1 enzyme, is involved in the regulation of anthocyanins and precursors of wide-range of pigmented polymers that gives attractive color to fruits and flowers of red grapes (*Vitis vinifera*) [27]. In addition the GT1 gene from *Cyclocarya paliurus* (Batalin) Iljinsk (a medicinal tree plant in China) is responsible for favoring substrate and biosynthesis of multiple bioactive compounds such as flavonoid glucosides and downstream pigmented compounds such as anthocyanidins [28]. GT1 is also involved in the catalyzation process by glucosylation of flavonols and flavones [28]. Moreover, the *Arabidopsis thaliana* UGT75C1 and UGT78D2 encode anthocyanin 5-O glucosyltransferase and flavonoid 3-O-glucosyltransferase enzymes respectively that are responsible for biosynthesis of anthocyanidins, pigmented compounds and flavonoids [29]. The overexpression of anthocyanin 3′-O-glucosyltransferase (3’GT) from Gentian (*Gentiana triflora*) in petunia showed accumulation of blue and purple color anthocyanins in the flower of transgenic petunia (*Petunia hybrida*) [30]. UGTs genes are directly involved anthocyanin and flavonol glycoside biosynthesis in *Arabidopsis thaliana* [31].

Based on previous published transcriptomic and gene expression studies on citrus [2, 13, 15, 32] we have selected key genes from the phenylprepanoid pathway; in addition, our gene expression and metabolic data showed that the sweet orange *Cs-UGT78D3* gene was differentially expressed (among the selected genes) and correlated with metabolic data (Additional file 1 and Additional file 2). So, we have chosen *Cs-UGT78D3* gene encoding *UDP-GLUCOSYL TRANSFERASE* from sweet orange (*Citrus sinensis*) to overexpress in *Arabidopsis thaliana* to evaluate its function. This study will help to understand the metabolic biosynthesis, regulation of genes and possible tolerance role of *UDP-GLUCOSYL TRANSFERASE* gene towards the stress; in addition, it also facilitates pharmacological industry and assists metabolic engineering to breed a cultivar with increased protective metabolites (phytochemicals) in citrus.

## Results

### Selection and overexpression UGTs gene

In *Citrus sinensis*, the UGTs genes regulations are positively correlated with flavonoids and anthocyanidins accumulation [33]. Additionally, citrus UGTs genes are involved in metabolism [34] and expressed during growth and development [35]. The citrus UGTs are involved in flavonoids biosynthesis [35, 36] and stimulates the anthocyanin biosynthesis in citrus [37]. Based on our (Additional file 2) and published transcriptome data [38], we have selected UGTs genes and confirmed that the UGT78D3 gene was differentially expressed in citrus specie (*Citrus sinensis*) by quantitative real time PCR (qRT-PCR) (Additional file 1). Thus, one UGTs gene was selected to overexpress in *Arabidopsis thaliana* to evaluate its function.

**Figure 1:**
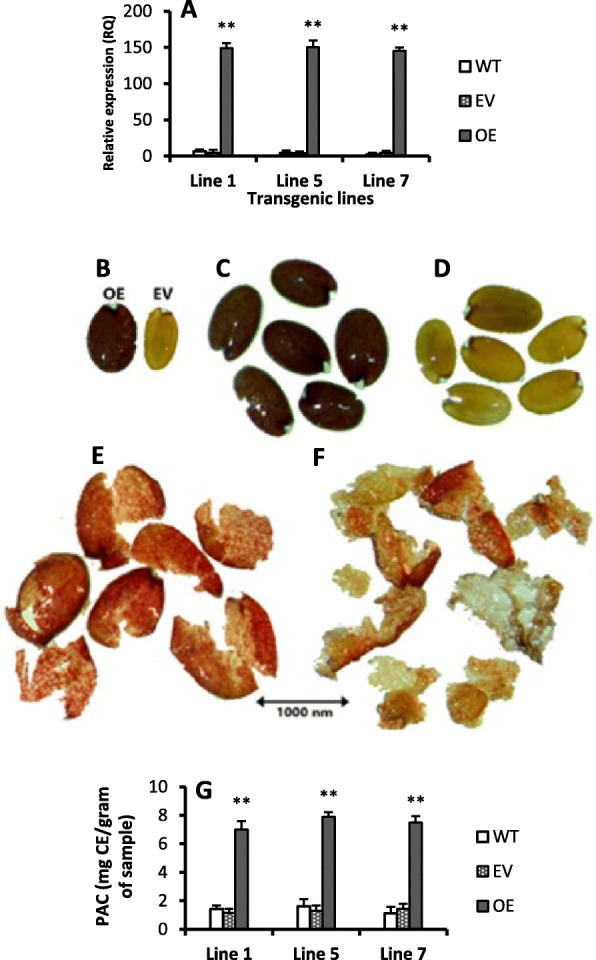
Overexpression of *Cs-UGT78D3* gene promotes proanthocyanidins accumulation in Arabidopsis. **a** Three individual lines showing the overexpression (OE) of *Cs-UGT78D3* gene (**b**) The OE and EV (empty vector) seed showed distinct color (**c**) OE seeds showing dark brown color (**d**) Wild type seeds showing pale yellow color (**e**) Vanillin assay of OE seed coats showing red color on each segment of seed coat (**f**) In wild type seed, only seed coats margins showing red color while the rest of seed coat color remained pale yellow (**g**) Proanthocyanidins were significantly increased in the seed coats. Values are mean of three replicates ± SE and Student’s t-test was used to compare 78D3-OE and WT, *p* < 0.01. (**) Highly significant. WT: Wild type, EV: Empty vector, OE: Overexpression

Transgenic Arabidopsis plants were selected at T_1_ generation, on MS + kanamycin medium for positive transgenic plant screening, and for further confirmation of transformed plants we did PCR by CaMV35S forward and reverse Cs5g24820 gene-specific primer. At T_2_ generation, three independent transgenic lines (OX-1, OX-5 and OX-7) were selected based on gene expression and PCR results. Furthermore, T_4_ generation was produced for light stress experiment. According to qRT-PCR results, the *Cs-UGT78D3* gene was more than 25 folds up-regulated in all transgenic lines as compared to wild type (Fig. 1a).

### Overexpression of *UDP-GLUCOSYL TRANSFERASE* gene promotes proanthocyanidins accumulation in seed coats

The seed coats color of transgenic Arabidopsis T_2_ lines becomes brownish as compared with empty vector seeds (Fig. 1b) and was also significantly differentiated from the wild type seed color which was pale yellow (Fig. 1d). At T_3_ and T_4_ generation, the seed coat color was stabilized i.e. dark brown (Fig. 1c). Generally, high accumulation of PAs in the Arabidopsis seed testa gives dark brown color to seed coats [39].

Vanillin reacts with leucoanthocyanidins monomers, terminal subunits of PAs, catechins and gives red color [40, 41]. Vanillin assay was conducted on T_3_ transgenic seeds. Interestingly, after vanillin treatment, the dark brown color of transgenic seed testa, showed distinct red color (Fig. 1e) as compared with wild type seeds (where only seed coat margins showed red color) (Fig. 1f). So, it is confirmed that the brown color of transgenic seed coats is due to PAs accumulation in seed testa (Fig. 1g) than wild type.

**Figure 2:**
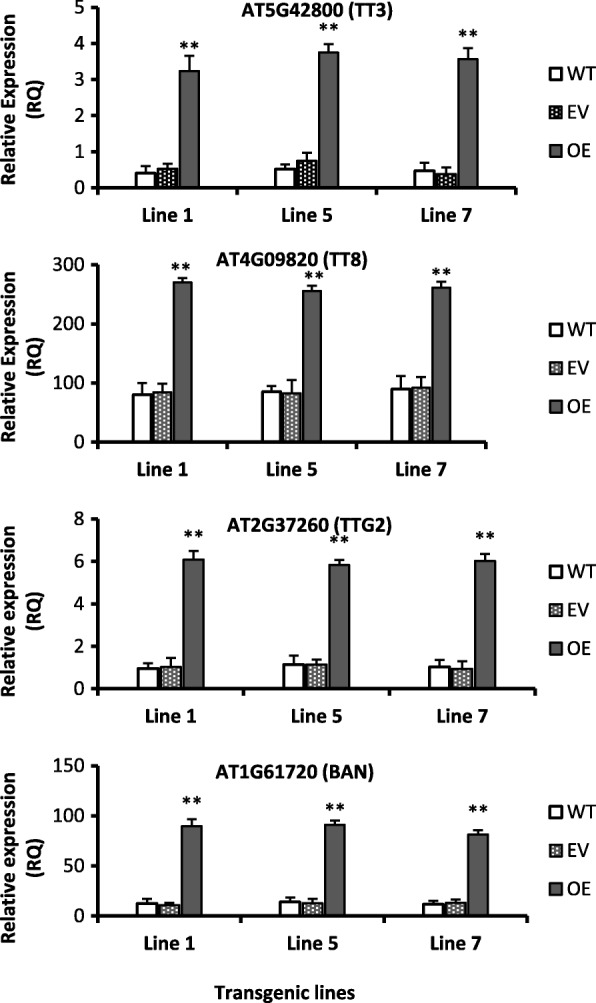
Genes involved in the regulation of anthocyanins and proanthocyanidins biosynthesis upregulated in the transgenic Arabidopsis without light stress. Values are mean of three replicates ± SE and Student’s t-test was used to compare 78D3-OE and WT, *p* < 0.01, (**) Highly significant. Gene IDs were taken from Arabidopsis genome website TAIR (https://www.arabidopsis.org/). TT3 or DFR: Dihydroflavonol 4-reductase; TT8: Transparent testa 8; TTG2: Transparent testa glabra 2; BAN: BANYULS. WT: Wild type, EV: Empty vector, OE: Overexpression

High concentration of proanthocyanidins contents (PAC) were observed in seeds of all transgenic lines than wild type (Fig. 1g). Additionally, gene related to proanthocyanidins biosynthesis pathway such as AT5G42800 (TT3), AT2G37260 (TTG2), AT4G09820 (TT8) and AT1G61720 (BAN) were significantly up-regulated (more than 4 folds) in transgenic seeds than wild type seeds (Fig. 2). Up-regulation of TTG2, TT8 and BAN during seeds formation will facilitates high accumulation of proanthocyanidins in the seeds coats of *Arabidopsis thaliana* [42, 43].

### High level of phenolic, flavonoid and anthocyanins in transgenic plants

**Figure 3:**
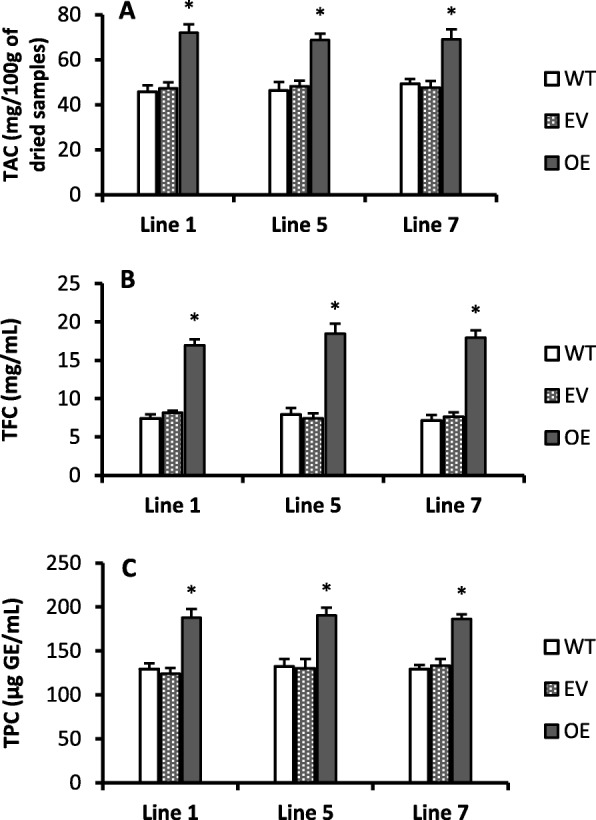
Anthocyanins, flavonoid and phenolic were significantly increased in transgenic lines without high light stress. **a**; Total anthocyanin contents (TAC) in leaves, **b**; Total flavonoid contents (TFC) in leaves, **c**; Total phenolic contents (TPC) in leaves. WT: Wild type, EV: Empty vector, OE: Overexpression. Values are mean of three replicates ± SE and Student’s t-test was used to compare 78D3-OE and WT, *p* < 0.05 (*) Significant; *p* < 0.01 (**) Highly significant

Higher concentration of total flavonoid (Fig. 3b) and total phenolic contents (Fig. 3c) were observed in transgenic lines as compared to wild type. According to qRT-PCR expression data, the *Cs-UGT78D3* gene was more than 25 folds (Fig. 1a) and some genes related to flavonols biosynthesis pathway such as AT3G55120 (TT5), AT3G51240 (TT6) and AT5G07990 (TT7) were induced 2–5 folds in all transgenic lines as compared with wild type (Additional file 3). TT5 is involved in the flavonoid and anthocyanin biosynthesis pathway [44]. The high concentration of total anthocyanin contents (TAC) (Fig. 3a) and total flavonoid contents (TFC) (Fig. 3b) in the leaves with significantly up-regulation of genes related to flavonoids pathways (Fig. 2) in all transgenic lines indicates that this gene is involved in increasing the total anthocyanin and flavonoid contents. Many plant phenolic and flavonoid compounds possess significant antioxidant properties that help plants to alleviate the free radical damage produced during light stress [17].

**Figure 4:**
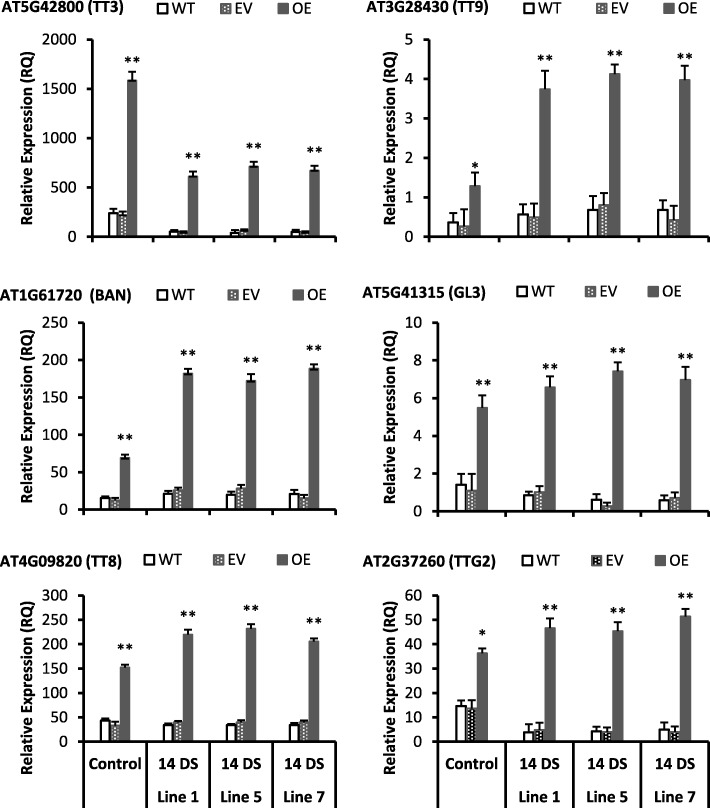
Genes involved in the regulation of flavonoids and anthocyanins biosynthesis were upregulated in the transgenic Arabidopsis after high light stress. Values are mean of three replicates ± SE and Student’s t-test was used to compare 78D3-OE and WT, *p* < 0.05 (*) Significant; *p* < 0.01 (**) Highly significant. 14 DS: 14 days light stress, WT: Wild type, EV: Empty vector, OE: Overexpression. Gene IDs were taken from Arabidopsis genome website TAIR (https://www.arabidopsis.org/)

Total anthocyanin contents (TAC) was significantly increased in all transgenic lines as compared with wild type (Fig. 3a). The TT3, TT8, AT3G28430 (TT9) and BAN were significantly induced (more than 4 folds) in the seeds and leaves of all transgenic lines as compared to wild type (Figs. 2 and 4). Moreover, AT5G41315 (GL3) and TTG2 were also significantly induced more than 4 folds in all transgenic lines than wild type. However, 2 fold or less than 2 fold increments has been observed in AT4G22880 (ANS), AT1G17260 (AHA) and AT5G05600 genes expression in transgenic lines than wild type (Additional file 3). The ANS, AHA and AT5G05600 genes are directly or indirectly involved in the anthocyanin biosynthesis (https://www.arabidopsis.org/). The TT3 and TT5 genes are involved in anthocyanin biosynthesis [44]. The metabolic and gene expression results revealed that *Cs-UGT78D3* gene is involved in inducing the flavonoid and anthocyanins biosynthesis pathway to accumulate more metabolites in transgenic Arabidopsis.

### Metabolic response of transgenic Arabidopsis against light stress

**Figure 5:**
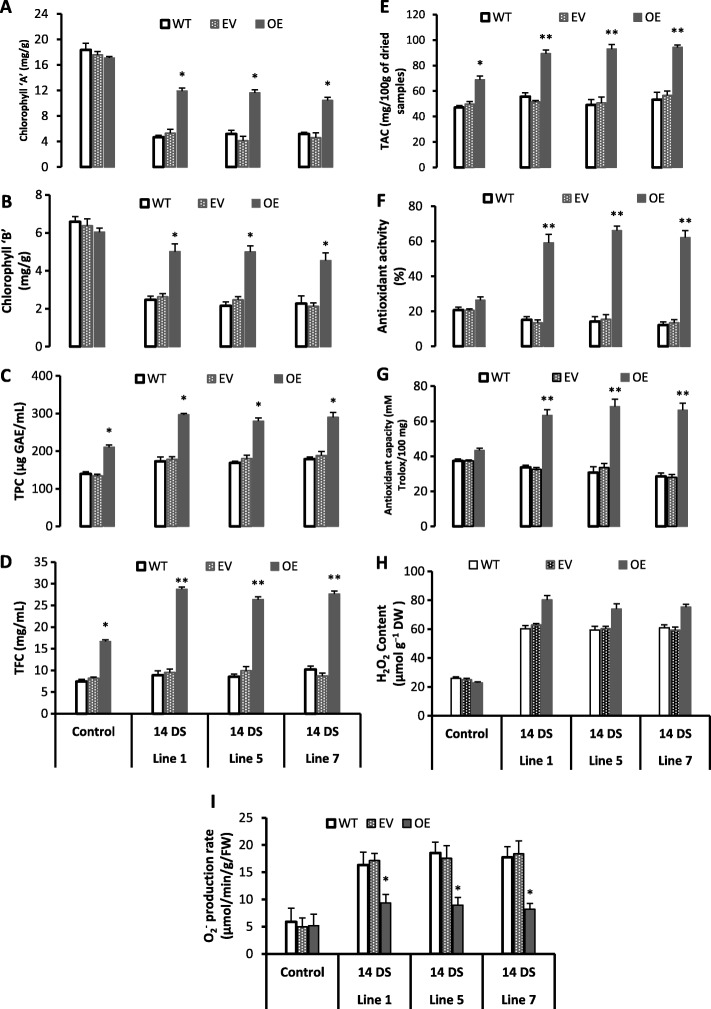
Effects of high light stress on antioxidant metabolism. **a**; Chlorophyll **a**, **b**; Chlorophyll **b**, **c**; Total phenolic contents (TPC), **d**; Total flavonoid contents (TFC), **e**; Total anthocyanin contents (TAC), **f**; Antioxidant activity, **g**; Antioxidant capacity, **h**; Hydrogen peroxide (H_2_O_2_ content), **i**; Superoxide radical (O_2_^−^). 14 DS: 14 days light stress, WT: Wild type, EV: Empty vector, OE: Overexpression. Control: Day 1; 14 DS: after 14 days light stress. Values are mean of three replicates ± SE and Student’s t-test was used to compare 78D3-OE and WT, *p* < 0.05 (*) Significant; *p* < 0.01 (**) Highly significant

**Figure 6:**
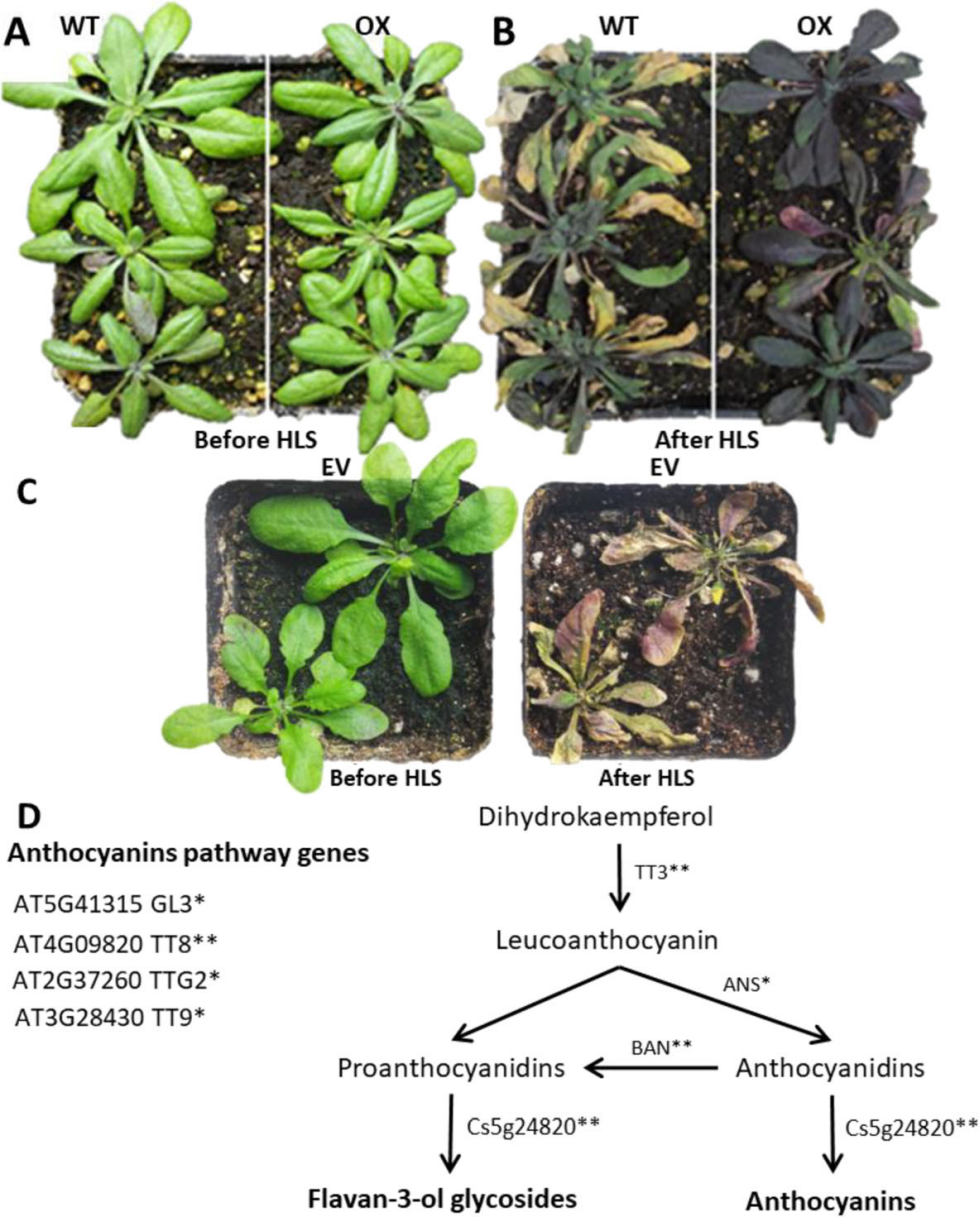
Phenotype of Arabidopsis plants after high light stress and the potential function step of citrus *Cs-UGT78D3* in anthocyanins biosynthesis pathway (**a**) 25 days old wild type (WT) and overexpressed (OX) Arabidopsis plants (**b**) After 14 days of high light stress WT and OX plants (**c**) HLS effects on empty vector (EV) lines (**d**) Assumed functional step of citrus *UDP-GLUCOSYL TRANSFERASE* gene (Cs5g24820) (Metabolic pathway has been made from KEGG www.genome.jp/kegg/pathway; TAIR https://www.arabidopsis.org/and [19]). Student’s t-test was used to compare 78D3-OE and WT, *p* < 0.05 (*) Significant; *p* < 0.01 (**) Highly significant. TT3 or DFR: Dihydroflavonol 4-reductase; TT8, TT9: Transparent Testa 8, 9; ANS: anthocyanidin synthase; BAN: Banyuls; TT9 and TTG2: triggers the accumulation of brown colored proanthocyanidins; GL3: TT8 and GL3 are basic helix-loop-helix (bHLHs) transcription factors that influence anthocyanin accumulation

TPC, TFC, TAC were increased significantly under 14 days high light stress (HLS) in all transgenic lines than wild type (Fig. 5c-e). In addition, all transgenic lines accumulated significantly high concentration of TAC than wild type after HLS (Fig. 5e). Moreover, all the transgenic lines showed dark purple color phenotype after HLS (Fig. 6b) whereas the wild type and empty vector leaves showed chlorosis and shrink phenotype (Fig. 6b, c). Additionally, after 14 days of light stress, all transgenic lines showed high expression of many genes related to anthocyanin biosynthesis pathway such as TT8, TT9, BAN (Fig. 4). Moreover, the TT3, TT8, TT9, BAN, TTG2 and GL3 genes (Fig. 4) were highly induced (2–15 folds) after high light stress in all transgenic lines and these genes are involved in proanthocyanidins and anthocyanins biosynthesis pathway (Fig. 6d). Anthocyanins are water soluble color pigment which has strong photoprotective properties and probably that is the phenomena behind the HLS tolerance of transgenic lines. Moreover, it was also previously reported that the anthocyanin compounds processes strong antioxidant properties and scavenging the free radicals produced during HLS [45]. So, our phenotypic, metabolic and gene expression data supports that flavonoids and anthocyanin were significantly accumulated in all transgenic lines after HLS.

Chlorophyll degradation was slower and antioxidant activity was higher in transgenic Arabidopsis in response to high light stress. Chlorophyll a and b were decreased dramatically in wild type and empty vector lines after 14 days of HLS; but all the transgenic lines maintained high level of chlorophyll a and b than wild type (Fig. 5a, b). Rapid degradation of chlorophyll, due to environmental stresses shows that the plants are more prone to oxidative stress with lower light harvesting capability [45, 46]. Our results showed rapid degradation of chlorophyll a and b with high level of superoxide radicals (Fig. 5i) in wild type plants while all transgenic lines possess high level of chlorophyll a and b with lower level of superoxide radicals (Fig. 5i) after 14 days of HLS; which clearly showed that wild type plants were under high stress and more prone to free radical damage than transgenic Arabidopsis lines (Fig. 5a).

The free radical scavenging potential during HLS has been examined in wild type and transgenic Arabidopsis lines. The antioxidant activity and capacity were extraordinarily higher in all transgenic lines after 14 days of HLS than wild type (Fig. 5f, g). In addition, the antioxidant activity and capacity were non-significantly reduced after 14 days of high light stress in wild type and empty vector lines than control (without stress) wild type and empty vector lines respectively (Fig. 5f, g). These results showed that the HLS transgenic Arabidopsis lines have high free radicals scavenging potential, as compared with wild type. Moreover, superoxide radicals were significantly lower in all transgenic lines after HLS than wild type (Fig. 5i). Abiotic stress tolerance is associated with high antioxidant activity and capacity [46]. Therefore, our results indicated that the wild type plants were underwent damage under high light stress due to high free radicals (Fig. 5i) whereas the transgenic lines show high antioxidant potential and are more tolerant to the stress.

The Hydrogen peroxide contents were increased in HLS transgenic lines than control transgenic lines and similar trend was observed in wild type plants (Fig. 5h). H_2_O_2_ is vital metabolite in plants that is involved in governing various defense responses against abnormal conditions such as cell signaling and sensing functions [47]. Recent researches have reported that H_2_O_2_ has been supposed to be involved in protecting plants during abiotic stresses by triggering the signal transduction pathways that facilities crops plant to acclimation in stressed environment [47, 48].

## Discussion

In *Arabidopsis thaliana,* UGTs gene expression are positively correlated with anthocyanin biosynthesis and flavonol glycoside accumulation [31]. The overexpression of glycosyl-transferase gene in *Citrus sinensis* revealed high accumulation of flavonoid glucosides and anthocyanidins biosynthesis [33]. Moreover, the UDP glucosyltransferase gene from *Citrus unshiu* (Marc.) is involved in metabolism and converts the limonoid aglycones into glucosides [34]. Delay expression of UGTs gene will delay the bitterness in citrus fruits [49]. The glucosyltransferase genes were significantly expressed during different growth and developmental stages of *Citrus paradisi* (cv. Duncan) and positively correlated with flavonoids biosynthesis [35]. The UGT708G1 from kumquat (*Fortunella crassifolia*) and UGT708G2 from satsuma mandarin (*Citrus unshiu*) are involved in flavonoids accumulation [36] and regulates the anthocyanin pigments biosynthesis [37] and in citrus UGTs genes respond to different environmental conditions [50, 51]. To the author’s knowledge, this study provided an evidence of UGT78D3s’ function in both anthocyanins and proanthocyanidins.

Accumulation of proanthocyanidins is governed by TT2, TT8, and TTG2 genes, and some downstream PAs biosynthetic genes and these genes are also involved in the flavonoid biosynthetic pathway [42]. Moreover, transcription factor such as TT8 regulates the UGTs genes to accumulate the proanthocyanidins and anthocyanins in Arabidopsis [42]. Generally, PAs were accumulated in the inner layer of seed testa and gives dark brown color to Arabidopsis seeds [42]. The terminal subunits of PAs react with vanillin and produces red color [40, 41]. PAs are colorless flavonoid polymers and PAs oxidation gives brown color to mature seeds [39]. In addition, PAs protect seeds from unfavorable environmental condition and enhance seed longevity [43]. The transgenic seeds showed dark brown color phenotype whereas wild type and empty vector seeds displayed pale yellow color (Fig. 1b-d). Moreover, vanillin assay showed accumulation of PAs in all transgenic seed coats (Fig. 1e). In addition, the proanthocyanidins contents were also high in seeds of all transgenic lines (Fig. 1g).

Our results showed that the TT8 and TTG2 both genes were up regulated in the transgenic lines as compared with wild type (Fig. 2). TT8 is a regulation factor which is involved in regulating the biosynthesis of flavonoids, proanthocyanidins and anthocyanins [42]. The brown color of Arabidopsis seeds are mainly due to PAs and their biosynthesis is governed by TT8 and TTG2 genes and these factors are involved in regulating the various biosynthetic gene of PAs pathway [42]. In addition, TT8 also regulates the JAZ (Jasmonate ZIM-domain) proteins to triggers the anthocyanin production. The TTG2 and TT8 expression are essential for BAN correct expression to accumulate PAs in seeds [42].

The Poinsettia (*Euphorbia pulcherrima*) leaves accumulates high level of water soluble anthocyanin pigments and withstand under high light stress, signifying the photo-defensive role of anthocyanin compounds [45]. Our results also showed high accumulation of anthocyanin content (Fig. 3a and 5e) and showed dark purple color in leaves of all transgenic lines (Fig. 6b) after HLS than wild type and empty vector (Fig. 6c); which demonstrate that overexpression of *Cs-UGT78D3* gene is involved in anthocyanin accumulation and protects plants from HLS damage. In addition, the real time PCR also showed many folds increment of TT8, TT9, BAN genes in all transgenic lines (Figs. 4 and 6d) and these genes are involved in the biosynthesis of anthocyanins.

The high light stress led to rapid degradation of chlorophyll a and b contents in the leaves of soybean (*Glycine max*) plants with decreased efficiency of photosynthetic apparatus [52]. In a recent study, similar results have been observed in Poinsettia (*Euphorbia pulcherrima*) plants when they were exposed to high light stress [45]. The plants who possess high chlorophyll contents and maintained high level of antioxidant activity and capacity can tolerate prolong abiotic stresses [53]. Our results also showed that high light stressed transgenic lines possesses significantly higher level of antioxidant activity (Fig. 5f, g), chlorophyll a and b contents (Fig. 5a, b) with lower superoxide radicals (Fig. 5i) than HLS wild type plants. Abiotic stress decreases the antioxidant potential, chlorophyll contents and also lowers the high harvesting ability of plants which ultimately triggers the production reactive oxygen species (ROS) and cause serve damage to cells (due to free radicals) [53].

Excessive light stress accumulates large amount of phenolic and flavonoid compounds such as flavonols in leaves, which has significant antioxidant capability to scavenging the ROS generated during abiotic [18, 23] and biotic stress [19]. Flavonols behaves as photo-protectants and possesses strong antioxidant activity that’s helps to counters the ROS species to fight against the oxidative stress caused by excessive light stress [17]. Additionally, the Poinsettia accumulates anthocyanins in leaves after HLS, that indicates strong photoprotective role of anthocyanins [45]. Flavonoids and anthocyanin holds resilient antioxidant properties and contributes to photoprotection [54]. Our metabolic results showed high level of anthocyanin and flavonoid contents in all transgenic lines compared to wild type (Fig. 3a, b). These outcomes showed that the overexpression of citrus *Cs-UGT78D3* gene is strongly linked with the flavonoids and anthocyanin biosynthesis; moreover, the gene expression data showed high expression of some genes involved in flavonol and anthocyanin biosynthesis (Fig. 4), that also supports the metabolic results (Fig. 3a, b, c). The transgenic plants become acclimatized to stress situation by triggering the flavonoids and anthocyanin biosynthesis and makes transgenic lines more tolerant against the HLS.

Recent researches have reported some positive and key roles of H_2_O_2_, by stimulating the complex signaling transduction pathways leading to systemic acquired resistance and stress acclimation under abiotic and biotic stresses [47, 48]. Moreover increment in H_2_O_2_ level will also induce some defense related genes [48]. Our results have showed high H_2_O_2_ contents in high light stress transgenic lines as compared with wild type (Fig. 5h). In addition, all transgenic lines showed high antioxidant activity and capacity (Fig. 5f, g), after HLS. This shows that transgenic lines possess better and strong free radical scavenging properties than wild type. This clearly demonstrates that under high light stress the transgenic lines showed less oxidative stress with better free radical scavenging capability than wild type Arabidopsis plants. Therefore, biochemical, metabolic, phenotypic and gene expression data supports that all transgenic lines were tolerant to high light stress; due to high level of anthocyanin and flavonoids with better antioxidant potential than wild type.

## Conclusion

Overexpression of *Cs-UGT78D3* showed dark brown color in seeds of all transgenic Arabidopsis lines due to proanthocyanidins accumulation. Moreover, all transgenic lines showed significantly high level of total anthocyanin and total flavonoid contents in leaves. After 14 days of high light stress, the transgenic Arabidopsis lines showed significant anthocyanins accumulation and maintained high antioxidant activity and capacity as compared with wild type. The gene expression data also supported the up-regulation of many flavonoids (flavonol) and anthocyanin biosynthesis genes in transgenic Arabidopsis lines than wild type. We demonstrate that overexpression of *Cs-UGT78D3* gene accumulates proanthocyanidins in transgenic seed coats and confers high light stress tolerance by accumulating total anthocyanin and flavonoid contents with improved antioxidant potential than wild type. This study also provided an ideal candidate gene for future metabolic engineering to breed a cultivar with increased protective metabolites (phytochemicals).

## Methods

### Plant materials and growth conditions

In this study, wild type *Arabidopsis thaliana* ecotype Columbia-0 (Col-0) (Arabidopsis seeds purchased online www.arashare.cn) was used to overexpress the *Citrus sinensis UDP-GLUCOSYL TRANSFERASE* (Cs5g24820) gene (citrus seeds were taken from citrus genomic lab in Huazhong Agricultural University). The wild type (WT) Arabidopsis seeds were sterilized with 70% (v/v) ethanol for 10 min followed by ethanol 100% (v) for 8 min, and then the seed were washed 4 times with double deionized water. Then the seeds were pour out on the Murashige and Skoog (MS) medium petri dishes plate containing 4.43 g MS (dried basal medium with vitamins supplement phyto-technology laboratories); 25 g sucrose; 10 g agar for 1 liter 1% (w/v) and petri plates were left in the growth chamber having 20–22 °C and then after 10 days the plants were transferred into the small pots (soil). Then the plants were grown in growth chamber for 3 weeks having an irradiance of 120 μmol quanta m^− 2^ per sec, with 70% relative humidity and temperature about 22 ± 3 °C, under 16/8 h light and dark period.

### Vector construction and agrobacterium transformation

The T-DNA Gateway technology (Invitrogen) pK7WG2D vector, was constructed (to overexpress the flavonol transferase gene of Citrus in Arabidopsis), which contains green fluorescent protein (GFP) and also confers kanamycin resistance (that helps for plant visual or manual selection) due to neomycin phosphotransferase II (nptII) gene [55]. By using cDNA, coding region of *Cs-UGT78D3* was amplified by means of PCR using gene specific primers, then the plasmid was extracted and first cloned into pDONR221 and then intervened by L. R clonase enzymatic reactions (invitrogen) (as prescribed by manufactures instructions) and cloned into binary Gateway Vector pK7WG2D. After that the pK7WG2D was transferred into GV3101 (Agrobacterium strain) and then transformed into the Arabidopsis, by dipping the wild type Arabidopsis flowers in Agrobacterium solution via floral dip method [56].

### Transgenic lines and light stress conditions

The transgenic Arabidopsis lines were developed via Agrobacterium-mediated transformation. The wild type (WT), transgenic Arabidopsis line 35S:PK7WG2D (empty vector), and three independent overexpressed 35S:PK7WG2D-UGT78D3 lines (OX-1, OX-5, OX-7) have been prepared by cross checking and selfing to get T_4_ stage transgenic plants for 14 days of light stress experiment. In each stage, the Arabidopsis seeds were first sown on MS medium having Kanamycin (50 mg/L) to get positive plants and later it was confirmed by qRT-PCR expression analysis. So, for light stress experiment, the 25 days old wild type, empty vector and transgenic plants were used and subjected to high light stress for 14 days in a growth camber with following conditions (Fig. 6a, c); 50,000 Lux light stress having 16 h light and 8 h dark, with 70% relative humidity and temperature about 24 ± 2 °C. On day 1 and after 14 days of HLS, the leaves samples were collected and immediately frozen by means of liquid nitrogen and preserved at − 80 °C for biochemical, metabolic and gene expression analysis.

### DNA extraction and PCR analysis

DNA was extracted by 2% CTAB method [57, 58]. Fifty to one hundred milligram (mg) fresh Arabidopsis leaves were crushed into fine powder and followed by addition of 700 μl of DNA buffer than 90 min incubation at 65 °C. After that 800 μl of chloroform-isoamyl alcohol (24:1) was added and then after 10 min of gentle inversions, centrifuged at 10,000 rpm for 10 min and DNA pellet was taken [58]. PCR was performed by using PCR-Master mix (dream tag green-Thermo Scientific) according to manufactures instructions.

### RNA extraction and quantitative real time PCR analysis

Fresh leaves were immediately frozen into liquid nitrogen after harvesting and then used for RNA extraction by using TRIzol RNA (extraction kit) reagent (Takara). RNA was extracted as described by manufacture’s instruction on TRIzol kit. After total RNA extraction, the complementary DNA (cDNA) was synthesized by using 1 μg of total RNA by means of (Vazyme, R223–01) HiScript II QRT (reverse transcriptase) SuperMix for qPCR (+gDNA wiper) methodology. After cDNA synthesis all the cDNA samples were stored at − 80 °C for further expression analysis (qPCR). For qRT-PCR was conducted by using SYBR Green (YEASEN Biotec. Co.Ltd.) PCR Master mix and all standard procedure were adopted as described by producer’s instructions. The qRT-PCR was done with three technical replicates (light cycler 480 multi-well plate 384-white) and performed by means of light cycler 480 II instrument (Roche). Relative expressions of pathway genes were calculated by means of 2 − ΔΔCt methodology [59]. Arabidopsis β-actin gene was used as an internal reference gene. The qRT-PCR primers details are documented in Additional file 4.

### Vanillin assay for proanthocyanidins determination

For vanillin assay, wild type and over-expressed seeds were taken in 1.5 ml of centrifuge tube followed by addition of I milliliter dye solution 1% vanillin (w/v) in 6 M of hydrochloric acid (HCl) [39]. About one layer of seeds has been taken to cover the bottom of 1.5 ml tube. Incubate the mature seeds for 1 hour at room temperature. After incubation, the seeds coats were gently separated with dissecting needle and tweezers on glass-slide by using stereomicroscope (OLYMPUS, SZ61 model). Then the light microscope (OLYMPUS, BX61 model) was used to photograph the stained seeds coats [60].

### Determination of proanthocyanidins contents (PAC)

Proanthocyanidins concentration was estimated by grinding 20 mg of Arabidopsis seeds with liquid nitrogen followed by adding 1 ml of extraction buffer (acetone 70%: water 29.5%: acetic acid 0.5%) with slightly modifications [61]. Then the samples were centrifuges at 4000 rpm and supernatant was taken for proanthocyanidins quantification. The reaction mixture contained 200 μl of above prepared solution followed by addition of 3 ml of 0.5% (w/v) vanillin dissolved in methanol and 1.5 ml of 4% (v/v) HCl. Then after 15 min the absorbance was taken at 550 nm on spectrophotometer (model UV-1800, Shimadzu corporation, Japan) whereas pure methanol was used as blank. The standard curve was generated by using catechin and the PAC value was expressed in milligrams of catechin equivalents (mg CE/gram of sample).

### Determination of chlorophyll content

For chlorophyll ‘a’ and ‘b’ estimation, the Arabidopsis leaves tissues (500 mg) were grounded into fine powder by using 10 ml (mL) of 80% (v/v) acetone [62], followed by 4 h of incubation at room temperature (RT) in dark. After that the sample tubes were centrifuged for 5 min at 12,000 rpm, and the supernatant was collected into a new tube then its absorbance was measured at 645 nm and 663 nm on spectrophotometer (80% (v/v) acetone was used as blank). Chlorophyll content was expressed in milligram per liter and calculated by using following formula:

$$ {\displaystyle \begin{array}{c}\mathrm{Chlorophyll}\ \mathrm{a}=\mathrm{OD}663\times 12.7-\mathrm{OD}645\times 2.69\left(\mathrm{mg}/\mathrm{L}\right),\\ {}\mathrm{Chlorophyll}\ \mathrm{b}=\mathrm{OD}645\times 22.9-\mathrm{OD}663\times 4.68\left(\mathrm{mg}/\mathrm{L}\right).\end{array}} $$

### Determination of total contents of phenolics and flavonoids

#### Extraction

100 mg of leaves tissues were crushed into powdered by using pestle and mortar followed by addition of 5 mL of 80% methanol and samples were left for 2 h at RT on an orbital shaker at 200 rpm followed by centrifugation [63]. The supernatant mixture was collected into a new tube while the remaining pellet was again extracted with the same procedure (with similar conditions) as described earlier and both supernatants were combined in 15 mL tube, and then used for estimation of total phenolics and total flavonoid contents.

#### Total phenolics contents (TPC)

Folin–Ciocalteu reagent (FCR) based methodology was used for determination of total phenolics content [63]. Three hundred microliter of above prepared extract was taken in a fresh 10 mL tube and mixed with (10-fold diluted FCR with distilled water) 2.25 mL of FCR followed by 5–10 gentle inversions and 5 min incubation at RT. Then I added 2.25 mL of sodium carbonate (Na_2_CO_3_) (60 g/L) solution into the reaction mixture. Then after 2 h of incubation at RT the absorbance was taken at 725 nm by means of spectrophotometer (model UV-1800, Shimadzu corporation, Japan). Standard curve was generated by using gallic acid (GA) and results were defined as milligram of GA-equivalents (GAE) per 1 gram of dried weight of plant leaves (mg GAE/g).

#### Determination of total flavonoid content (TFC)

Total flavonoid content was measured by means of colorimetric method with minor modification [64]. About 500 μl of the prepared methanolic extract was taken into 10 mL new tube followed by addition of 2.25 mL of distilled water and mix well then added 150 μl of 5% sodium nitrite (NaNO_2_) solution followed by 6 min incubation at RT. After that 300 μl solution of 10% aluminum chloride hexahydrate (AlCl_3_·6H_2_0) was added into the reaction mixture with 5 min of incubation at RT and then 1000 μl of 1 Molar (M) sodium hydroxide (NaOH) was added followed by vertex for 30 s. The reaction mixture absorbance was taken instantly at 510 nm by means of spectrophotometer (model UV-1800, Shimadzu corporation, Japan). Standard curve was generated by using rutin compound and results were defined as milligram rutin equivalents (RE) per 1 gram of dried plant leaves sample (mg RE/g).

### Total anthocyanin contents (TAC)

For determination of total anthocyanin content 100 mg of leaves tissues were grounded (using liquid nitrogen) by means of mortar and pestle [65, 66]. After that the samples were re-suspended in five volumes (based on fresh weight) of extraction solution (having 45% methanol (v/v) and 5% acetic acid v/v) followed by gentle inversion and then centrifuged at (10,000 rpm) for 10 min at RT. The supernatant solution was collected into a new tube to check the absorbance at 530 nm and 657 nm by anthocyanin measurement and chlorophylls respectively thru spectrophotometer (model UV-1800, Shimadzu corporation, Japan). Then by using the following formula the anthocyanin contents were measured by correction in the 530 nm absorbance by chlorophylls:

$$ {\mathrm{TAC}}_{\left(\mathbf{mg}/\mathbf{10}\mathbf{0g}\ \mathbf{of}\ \mathbf{dried}\ \mathbf{weight}\right)}=\Big(\mathrm{absorbance}\ \mathrm{at}\ 530\ \mathrm{nm}-\left(0.25\times \mathrm{absorbance}\ \mathrm{at}\ 657\ \mathrm{nm}\right)\times \mathrm{extraction}\ \mathrm{volume}\ \left(\mathrm{mL}\right)\times 1/\mathrm{weight}\ \mathrm{of}\ \mathrm{leave}\ \mathrm{tissue}\ \mathrm{sample}\ \left(\mathrm{g}\right). $$

*For anthocyanin we used 5 times extraction volume and 0.1 g leave tissue sample.

### Antioxidant capacity and activity (DPPH free radical scavenging assay)

For antioxidant capacity and activity fresh Arabidopsis leaves were ground (100 mg) and homogenized in 1 mL of extraction solution (ethanol, water, and acetic acid, 70, 29, and 1% respectively) and then centrifuged [67] with slight modifications. The supernatant was used to calculate antioxidant capacity by using the 30 μl of above prepared solution followed by addition of 2.97 mL of 0.1-mM 2,2-diphenyl-1-picrylhydrazyl (DPPH) followed by 30 min incubation in dark (in RT). Then the sample absorbance was taken at 517 nm by means of spectrophotometer (model UV-1800, Shimadzu corporation, Japan). Thirty microliter of extraction solution (without plant sample) in 2.97 mL of DPPH is used as control. The antioxidant capacity was calculated by generating standard curve of trolox and samples were expressed in (mM Trolox/ 100 mg). While the antioxidant (free radical scavenging) activity is described by using the following formula:

$$ \mathrm{Antioxidant}\ \mathrm{activity}\ \left(\%\right)=\left[1-\left\{\ \mathrm{sample}\ \mathrm{OD}/\mathrm{control}\ \mathrm{OD}\right\}\right]\times 100. $$

### Hydrogen peroxide (H_2_O_2_)

Hydrogen peroxide was measured by using trichloro-acetic acid (TCA) method [68]. Ground leaves samples (0.1 g) were re-suspended in 1000 μl of TCA (0.1%) solution in an ice bath and then centrifuged for 10 min at 10,000 rpm. The 500 μl of supernatant was taken into a new tube and added 500 μl of 10-mM potassium phosphate buffer followed by addition of 1000 μl of 1 M potassium iodide (KI) then mix well and checked the absorbance reading at 390 nm by means of spectrophotometer (model UV-1800, Shimadzu corporation, Japan). The sample absorbance was calculated by comparing the standard curve absorbance of commercial H_2_O_2_. The H_2_O_2_ contents were expressed in micromoles/g of dried samples.

### Superoxide radical’s determination

The superoxide radicals (O_2_^**−**^) were measured in 0.1 g of fresh leaves tissues [69]. The one unit of superoxide radical was defined as 0.1 units change in absorbance, per min at the corresponding wavelength values. The standard curve was generated by using nitrite ion (NO_2_^−^). The absorbance was measured at 530 nm on spectrophotometer (model UV-1800, Shimadzu corporation, Japan).

### Statistical analysis

The Statistix 8.1 (Tallahassee Florida, USA) statistical package was used to analyze the data. The standard error and graphs were made by using Microsoft Excel 2010 program (Microsoft Corp., Redmond, WA, USA). Differences were considered significant at *p* < 0.05 and highly significant at *p* < 0.01.

## Supplementary information

**Additional file 1: Table S1**. Showing qPCR primer sequence used in this study for gene expression analysis. **Figure S1.** Showing the gene expression pattern in citrus species at different stages. SWO: *Sweet orange*; AB: *Atalantia buxifolia*; CG: *Citrus grandis*. **Figure S2**. Representing the gene expression results under drought and High light stress on *Citrus sinensis* leaves. 14 HLS, 14 days of high light stress; 14DDS: after 14 days of drought stress. Values are mean of three replicates ± SE and Student’s t-test was used to compare control and stressed plants *p* < 0.05. (*) Significant: *P* < 0.01 (**) highly significant. **Table S2.** Showing the details and mode of metabolites used in this study. **Figure S3.** Heat map and hierarchical cluster analysis (HCA) using the square of peaks of detected metabolites in different citrus germplasm (AB; *Atalantia buxifolia*, CG; *Citrus grandis*, and CS; *Citrus sinensis*). Column represents varieties and row characterized flavonoids and anthocyanins.

**Additional file 2: **Raw transcriptomic data of 12 key genes: Representing the gene expression pattern data in three citrus species at different stages of development (in seed, leaf, flower and fruit). SWO: *Sweet orange*; AB: *Atalantia buxifolia*; CG: *Citrus grandis*.

**Additional file 3: Figure S1.** Showing the expression of different genes used in this study. WT: Wild type, EV: Empty vector, OE: Overexpression. Values are mean of three replicates ± SE and Student’s t-test was used to compare 78D3-OE and WT, *p* < 0.05 (*) Significant; *p* < 0.01 (**) Highly significant. Gene IDs were taken from Arabidopsis genome website TAIR (https://www.arabidopsis.org/).

**Additional file 4: Table S1.** Showing the gene ID of transcription factor and enzyme that involved in the flavonoid and anthocyanin pathway and their primers sequence used for qRT-PCR.

## Data Availability

All data generated or analyzed during this study are included in this published article [and its supplementary information files].

## Abbreviations

CE: Catechin equivalents
DPPH: 2,2-diphenyl-1-picrylhydrazyl
FCR: Folin–Ciocalteu reagent
G: gram
GA: Gallic acid
H_2_O_2_: Hydrogen peroxide
HLS: High light stress
M: Molar
Mg: milligram
mL: Milliliter
O_2_^**−**^: Superoxide radicals
OX: Overexpression
PAC: Proanthocyanidins contents
PAs: Proanthocyanidins
qRT-PCR: Quantitative real time PCR
ROS: Reactive oxygen species
rpm: Rounds per minute
RT: Room temperature
TAC: Total anthocyanin contents
TFC: Total flavonoid content
TPC: Total phenolics contents
UGTs: UDP glycosyl-transferase
μg: microgram
