# Supplementary material for: Ectopic expression of citrus UDP-GLUCOSYL TRANSFERASE gene enhances anthocyanin and proanthocyanidins contents and confers high light tolerance in Arabidopsis
Source: BMC Plant Biol. 2019 Dec 30;19:603. doi: 10.1186/s12870-019-2212-1 (PMC6937997; doi:10.1186/s12870-019-2212-1)
Supplement: Supplementary file 1 — Additional file 1: Table S1. Showing qPCR primer sequence used in this study for gene expression analysis. Figure S1. Showing the gene expression pattern in citrus species at different stages. SWO: Sweet orange; AB: Atalantia buxifolia; CG: Citrus grandis. Figure S2. Representing the gene expression results under drought and High light stress on Citrus sinensis leaves. 14 HLS, 14 days of high light stress; 14DDS: after 14 days of drought stress. Values are mean of three replicates ± SE and Student’s t-test was used to compare control and stressed plants p < 0.05. (*) Significant: P < 0.01 (**) highly significant. Table S2. Showing the details and mode of metabolites used in this study. Figure S3. Heat map and hierarchical cluster analysis (HCA) using the square of peaks of detected metabolites in different citrus germplasm (AB; Atalantia buxifolia, CG; Citrus grandis, and CS; Citrus sinensis). Column represents varieties and row characterized flavonoids and anthocyanins. [file 12870_2019_2212_MOESM1_ESM.docx]

| **Serial No.** | **Gene Code** | **Gene annotation** | **Arabidopsis Gene ID** | ***C. sinensis***  **Gene ID** | **Primer Sequence 5’ to 3’** |
| --- | --- | --- | --- | --- | --- |
| **1** | PAL1 | cinnamic acid biosynthetic process | At2g37040 | Cs6g11940 | F: ACATTGCCGGACTGCTCAC  R: AACCCGAAACCCGCTTG |
| **2** | C4H | phenylpropanoid metabolic process | At1g65060 | Cs5g24900 | F: ACTTACTCTTATGCCGAAACGC  R: TGGAAGCTCCCATGAACGA |
| **3** | CHS | flavonoid biosynthetic process | At5g13930 | Cs2g14720 | F: CTTTGTTCGGTGATGGTGCT  R: CCGTCAGAGTCAGGGAGGAT |
| **4** | CHI | flavonoid biosynthetic process | At3g55120 | Cs7g28130 | F: GAGGATTGGAGATTGAAGGGAA  R: ACACTCCTATCGCCGTGAACT |
| **5** | F3’H (CYP75B1) | flavonoid biosynthetic process | At5g07990 | Cs5g11730 | F: TGGCGGATGCTGAGGAA  R: GGGCATTCACAACGCACA |
| **6** | FLS1 | flavonoid biosynthetic process | At5g08640 | Cs1g19280 | F: CTTTCTGCCCTCACCGTTCT  R: CATTCTCGTCTTGTCCTTGCTAA |
| **7** | DFR | anthocyanin-containing compound biosynthetic process | At5g42800 | Cs3g25090 | F: TGGCTATGCTGTTCGTGCTAC  R: TTCCCTCTTCGGCTAAATCG |
| **8** | LDOX/ANS | anthocyanin-containing compound biosynthetic process, | At4g22880 | Cs5g09970 | F: CTTCGGGAAAGATTCAGGGTT'  R: TCGTCGCTAGGCTTCTCAGTT |
| **9** | ANR/BAN | negative regulation of flavonoid biosynthetic process, | At1g61720 | Cs2g07290 | F: CAATCCACCCAAAAGAAGACG  R: ACAGTAGCATGAACAGCATAGC |
| **10** | F3RhaT (UGT78D1) | flavonol biosynthetic process, | At1g30530 | Cs5g24820 | F: TATCAGGGACAAAATAGGCACC  R: TCAGGCAAGTCGGCGACGCGTA |
| **11** | F3GlcT (UGT78D2) | phenylpropanoid metabolic process | At5g17050 | Cs5g24820.1 | F: TATCAGGGACAAAATAGGCACC  R: AACTCCTTCAGGCAAGTCGG |
| **12** | F3AraT (UGT78D3) | Anthocyanidin 3-O-glucosyltransferase 2 | At5g17030 | Cs5g24820 | F: GACAAAATAGGCACCCAAAGTC  R: AACTCCTTCAGGCAAGTCGG |

Additional file 1

We have selected the key gene from flavonoids pathway in plants (Table 1). Then we have taken the gene expression pattern of selected genes (transcriptomic data), the *UGT78D3* was highly expressed in sweet orange as compared with *Atalantia buxifolia* (Primitive citrus) and *Citrus grandis* (Pumelo) (Figure 1) corresponding to the metabolic data (Table 2 and Figure 3). Then we grow the sweet orange seedlings and exposed them to high light stress and drought stress. The *CsUGT78D3* was high expressed after high light stress (Figure 2) so, we have cloned the *CsUGT78D3* gene from sweet orange and overexpressed it in *Arabidopsis thaliana* to evaluate its function and possible role in stress.

Table S1 Showing qPCR primer sequence used in this study for gene expression analysis.

**Figure S1** Showing the gene expression pattern in citrus species at different stages. SWO: Sweet orange; AB: *Atalantia buxifolia*; CG: *Citrus grandis*.

**Figure S2** Representing the gene expression results under drought and High light stress on *Citrus sinensis* leaves. 14 HLS, 14 days of high light stress; 14DDS: after 14 days of drought stress. Values are mean of three replicates ± SE and Student’s t-test was used to compare control and stressed plants p < 0.05. (*) Significant: P < 0.01 (**) highly significant.

**Table S2** Showing the details and mode of metabolites used in this study.

| **Sr No** | **Index** | **Ion mode** | **Molecular Weight (Da)** | **Ionization model** | **KEGG ID** | **Compounds** | **Class** |
| --- | --- | --- | --- | --- | --- | --- | --- |
| 1 | Cit777 | Positive | 580.1792 | [M+H]+ | C09793 | Narirutin | Flavanone |
| 2 | Cit778 | Positive | 580.1792 | [M+H]+ | C09789 | Naringin | Flavanone |
| 3 | Cit781 | Positive | - | [M+H]+ | - | Xanthotol | Coumarins |
| 4 | Cit782 | Positive | 216.0423 | [M+H]+ | C01557 | Bergapten | Coumarins |
| 5 | Cit783 | Positive | 186.0317 | [M+H]+ | C09305 | Psoralen | Coumarins |
| 6 | Cit785 | Positive | 178.0266 | [M+H]+ | C03093 | Daphnetin | Coumarins |
| 7 | Cit786 | Positive | 176.0473 | [M+H]+ | C09268 | Herniarin | Coumarins |
| 8 | Cit788 | Positive | 354.0951 | [M+H]+ | C01527 | Scopolin | Coumarins |
| 9 | Cit326 | Positive | 463.123 | Protonated | - | Peonidin O-hexoside | Anthocyanins |
| 10 | Cit632 | Positive | 330.1 | [M+H]+ | - | Tricin | Flavone |
| 11 | Cit356 | Positive | 477.1 | Protonated | - | Rosinidin O-hexoside | Anthocyanins |
| 12 | Cit463 | Positive | 301.1 | Protonated | C08726 | Peonidin | Anthocyanins |
| 13 | Cit1031 | Negative | 466.1 | [M-H]- | - | Cyanidin O-syringic acid | Anthocyanins |
| 14 | Cit1069 | Negative | 490.1 | [M-H]- | - | Cyanidin O-acetylhexoside | Anthocyanins |
| 15 | Cit1263 | Negative | 286 | [M-H]- | C01514 | Luteolin | Flavone |
| 16 | Cit1291 | Negative | 286.048 | [M-H]- | C05903 | Kaempferol | Flavonol |
| 17 | Cit1265 | Negative | 302.043 | [M-H]- | C00389 | Quercetin | Flavonol |
| 18 | Cit688 | Positive | 254.0579 | [M+H]+ | C10028 | Chrysin | Flavone |
| 19 | Cit1185 | Negative | 580.1792 | [M-H]- | C09789 | Naringenin 7-O-neohesperidoside (Naringin) | Flavanone |
| 20 | Cit631 | Positive | 300.0634 | [M+H]+ | C04293 | Chrysoeriol | Flavone |
| 21 | Cit1287 | Negative | 272.0685 | [M-H]- | C00509 | Naringenin | Flavanone |
| 22 | Cit627 | Positive | 270.0528 | [M+H]+ | C01477 | Apigenin | Flavone |
| 23 | Cit555 | Positive | 176.0473 | [M+H]+ | C03081 | 4-Methylumbelliferone | Coumarins |
| 24 | Cit484 | Positive | 576.1268 | [M+H]+ | C10237 | Procyanidin A2 | Proanthocyanidins |
| 25 | Cit302 | Positive | 493 | Protonated | - | Malvidin 3-O-galactoside | Anthocyanins |
| 26 | Cit309 | Positive | 493.2 | Protonated | C12140 | Malvidin 3-O-glucoside (Oenin) | Anthocyanins |
| 27 | Cit1284 | Negative | 274.084 | [M-H]- | C00774 | Phloretin | Flavanone |
| 28 | Cit225 | Positive | 465.1 | Protonated | C12138 | Delphinidin 3-O-glucoside (Mirtillin) | Anthocyanins |
| 29 | Cit693 | Positive | 402.132 | [M+H]+ | C10112 | Nobiletin | Flavone |
| 30 | Cit709 | Positive | 372.121 | [M+H]+ | C10190 | Tangeretin | Flavone |
| 31 | Cit1259 | Negative | 288.063 | [M-H]- | C05631 | Eriodictyol | Flavanone |
| 32 | Cit222 | Positive | 611 | Protonated | C08639 | Cyanidin 3,5-O-diglucoside (Cyanin) | Anthocyanins |
| 33 | Cit1302 | Negative | 302.079 | [M-H]- | C01709 | Hesperetin | Flavanone |
| 34 | Cit1193 | Negative | 610.19 | [M-H]- | C09755 | Hesperetin 7-rutinoside (Hesperidin) | Flavanone |
| 35 | Cit619 | Positive | 272.069 | [M+H]+ | C06561 | Naringenin chalcone | Flavanone |
| 36 | Cit633 | Positive | 148.052 | [M+H]+ | C02274 | 3,4-Dihydrocoumarin | Coumarins |
| 37 | Cit1194 | Negative | 192.042 | [M-H]- | C01752 | Scopoletin (7-Hydroxy-5-methoxycoumarin) | Coumarins |
| 38 | Cit1231 | Negative | 302.043 | [M-H]- | C10192 | Tricetin | Flavone |
| 39 | Cit652 | Positive | 160.052 | [M+H]+ | - | 6-MethylCoumarin | Coumarins |
| 40 | Cit1299 | Negative | 346.069 | [M-H]- | C11620 | Syringetin | Flavonol |
| 41 | Cit347 | Positive | 178.027 | [M+H]+ | C09263 | Esculetin (6,7-dihydroxycoumarin) | Coumarins |
| 42 | Cit388 | Positive | 208.037 | [M+H]+ | C09265 | 6-Methoxy-7,8-DihydroxyCoumarin | Coumarins |
| 43 | Cit267 | Positive | 340.079 | [M+H]+ | C09264 | Esculin (6,7-Dihydroxycoumarin-6-glucoside) | Coumarins |
| 44 | Cit1294 | Negative | 302.2788 | [M-H]- | C09756 | Homoeriodictyol | Flavanone |
| 45 | Cit1288 | Negative | 272.069 | [M-H]- | C09614 | Butin | Flavone |
| 46 | Cit580 | Positive | 206.058 | [M+H]+ | C09311 | Scoparone | Coumarins |
| 47 | Cit389 | Positive | 287.24 | Protonated | C05905 | Cyanidin | Anthocyanins |


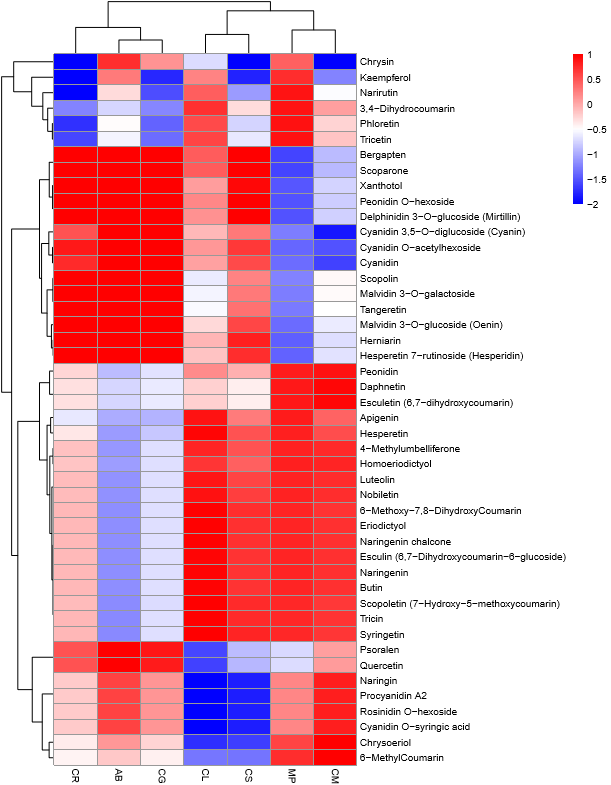


**Figure S3.** Heat map and hierarchical cluster analysis (HCA) using the square of peaks of detected metabolites in different citrus germplasm germplasm (AB; *Atalantia buxifolia*, CG; *Citrus grandis*, and CS; *Citrus sinensis*). Column represents varieties and row characterized flavonoids and anthocyanins. Other abbreviations: MP; *Murraya paniculata*, CL; *Citrus latipes*, CM; *Citrus medica*, CR; *Citrus reticulata*.
