# Supplementary material for: Ectopic expression of citrus UDP-GLUCOSYL TRANSFERASE gene enhances anthocyanin and proanthocyanidins contents and confers high light tolerance in Arabidopsis
Source: BMC Plant Biol. 2019 Dec 30;19:603. doi: 10.1186/s12870-019-2212-1 (PMC6937997; doi:10.1186/s12870-019-2212-1)
Supplement: Supplementary file 3 — Additional file 3: Figure S1. Showing the expression of different genes used in this study. WT: Wild type, EV: Empty vector, OE: Overexpression. Values are mean of three replicates ± SE and Student’s t-test was used to compare 78D3-OE and WT, p < 0.05 (*) Significant; p < 0.01 (**) Highly significant. Gene IDs were taken from Arabidopsis genome website TAIR (https://www.arabidopsis.org/). [file 12870_2019_2212_MOESM3_ESM.docx]

Additional file 3

Figure S1: Showing the expression of different genes used in this study. WT: Wild type, EV: Empty vector, OE: Overexpression. Values are mean of three replicates ± SE and Student’s t-test was used to compare 78D3-OE and WT, p < 0.05 (*) Significant; p < 0.01 (**) Highly significant. Gene IDs were taken from Arabidopsis genome website TAIR (<https://www.arabidopsis.org/>).
