# Supplementary material for: Ectopic expression of citrus UDP-GLUCOSYL TRANSFERASE gene enhances anthocyanin and proanthocyanidins contents and confers high light tolerance in Arabidopsis
Source: BMC Plant Biol. 2019 Dec 30;19:603. doi: 10.1186/s12870-019-2212-1 (PMC6937997; doi:10.1186/s12870-019-2212-1)
Supplement: Supplementary file 4 — Additional file 4: Table S1. Showing the gene ID of transcription factor and enzyme that involved in the flavonoid and anthocyanin pathway and their primers sequence used for qRT-PCR. [file 12870_2019_2212_MOESM4_ESM.docx]

**Additional file 4**

**Table S1.** Showing the gene ID of transcription factor and enzyme that involved in the flavonoid and anthocyanin pathway and their primers sequence used for qRT-PCR.

| **Serial no.** | **Enzyme codes** | **Description** | **Arabidopsis gene ID** | **Primer seq. used for q-PCR (5’ to 3’)** |
| --- | --- | --- | --- | --- |
| 1 | Cs F | UDP-glucosyl transferase 78D3 |  | GATGAAAGCGTCGTGGGTT |
| 1.1 | Cs R | Cs5g24820 |  | ATGAGCAGTGAGCGAGGTTG |
| 2 | UDP F | UDP-glucosyl transferase 78D3 | AT5G17030 | GATGAAAGCGTCGTGGGTT |
| 2.1 | UDP R |  |  | ATGAGCAGTGAGCGAGGTTG |
| 3 | TT3 F | DFR (Transparent testa 3) | AT5G42800 | CCAAACGCCAAGACGCTAC |
| 3.1 | TT3 R |  |  | TTCACTGTCGGCTTTATCACTTC |
| 4 | TT4 F | Transparent testa 4 | AT5G13930 | CGGTCAGGCTCTTTTCAGTG |
| 4.1 | TT5 R |  |  | CCTCAAATGTCCGTCTATGGC |
| 5 | TT5 F | Transparent testa 5 | AT3G55120 | TTTGTACCGTCCGTCAAGTCA |
| 5.1 | TT5 R |  |  | ATTCTGTTAGCTCCTCCGTAGTTT |
| 6 | TT6 F | Flavanone 3-hydroxylase | AT3G51240 | ACACTGACCCTGGAACCATTAC' |
| 6.1 | TT6 R |  |  | ATCAGCATTCTTGAACCTCCC |
| 7 | TT7 F | Flavonoid 3’-monooxygenase | AT5G07990 | ATCATCGGAAACCTCCCTCA |
| 7.1 | TT7 R |  |  | CGAAATTGGCGTCGTGTATT |
| 8 | TT8 F | Stimulates proanthocyanidin and anthocyanin biosynthesis | AT4G09820 | TCCGAGCAGAACTACCAACG |
| 8.1 | TT8 R |  |  | GCCGCCCCTTCAACAAA |
| 9 | TT9 F | Transparent testa 9 | AT3G28430 | CGAGCAGCCGCCTATTTAT |
| 9.1 | TT9 R |  |  | TTCAGTTCGGTGCCAGGTT |
| 10 | BAN F | Banyuls | AT1G61720 | ATGCAGAAGCTATCTGGCTCG |
| 10.1 | BAN R |  |  | TCCGCAAGAAACAAATGGG |
| 11 | JOX2 F | Jasmonate-induced oxygenase2 | AT5G05600 | GACCAACATCCCAATCATAGACC |
| 11.1 | JOX2 R |  |  | TTCACCACCTGGAAGAACCC |
| 12 | GL3 F | Encodes a basic helix loop helix domain protein | AT5G41315 | AAGACTAGGGCAAATTCAAGAGC |
| 12.1 | GL3 R |  |  | GCGGTCCGAGAATTAACTGAT |
| 13 | ANS F | Anthocyanidin synthase (LDOX) | AT4G22880 | ATCGTGGGTTGGTGAATAAGG |
| 13.1 | ANS R |  |  | TTGAGCAAAAGTCCGTGGAG |
| 14 | AHA10 F | Transparent testa 13 | AT1G17260 | CAACAGGGTGCCATTACGAA |
| 14.1 | AHA10 R |  |  | GGTTCCAGTTTTATCACAGCAGA |
| 15 | TTG1 F | TRANSPARENT TESTA GLABRA 1 | AT5G24520 | GGAAACCGCCGTCACATAC |
| 15.1 | TTG1 R |  |  | GTCATTGAATCGGAATCGAAAG |
| 16 | AT-Actin 1 | Internal reference control |  | GGAAGGATCTGTACGGTAAC |
| 16.1 | AT-Actin 2 |  |  | TGTGAACGATTCCTGGACCT |
